# Supplementary material for: Chromothripsis during telomere crisis is independent of NHEJ, and consistent with a replicative origin
Source: Genome Res. 2019 May;29(5):737–49. doi: 10.1101/gr.240705.118 (PMC6499312; doi:10.1101/gr.240705.118)
Supplement: Supplemental Material [file supp_gr.240705.118_Supplemental_file_1.zip › contigs/annotated_contigs/DB53/contig.2.DB53_length_583_mean_cov_4.56946826758.docx]

**DB53_length_583_mean_cov_4.56946826758**

```
TCTCTAACATTCATGTATGAGTTGGCTGTTCATATGGATCAATATATCACTTATTACCCAATCCTTTAAGGCTTGATTTATTTTCCTTT
 >chr8:135846222-135846560 + E=1e-188 p=1e-02
CCCTTCAGATTGTAAAACTCTTGAATGGCAGGAACCCTGTCTAGTTCCACATCGAATTTCCAGCCTGTTCCTCAGTAACAGCACCACTC

AACAACTAGCTATTGAATGGCTGAGTGAGCTCCCTGCCTATCTTATAAAATTATTAGGGGCAAACTTTATTTTTATATTTATATCTCAA

TACCAGGAATTTTCTGGGAAGTTAAGAATTTCCAATAAATCTTGGACTACTCTAAAATGCACCAGA|AATTA|AGTTGTATGATTTTTC
                                                                   >chr8:135482087-1354
TCAAGCTTGCAAAAGCAATTAATGCTTCCCGCCCCAGCCAGGGGTGGGGACTCTTTGGTTCAGCTGCCTTCCTGGCGAGGCTAGGGAAC
82337 - E=9e-139
GTCCTAAAATTCAGACTCTATGTTGGTATTTCTGAGCTCCACAGATCCCACCCCTCACACTCCAATAGATCCACTCAACCCCCAGCTCC

GGTCAGGGTGGGATGTGAATTTCGACCCAGATTTTCCTGTCCCTACCTGTG
```
